# Bi-dimensional acculturation and depressive symptom trajectories from pregnancy to 1 year postpartum in marriage-based immigrant women in Taiwan

**DOI:** 10.1017/S0033291720004195

**Published:** 2022-09

**Authors:** Hung-Hui Chen, Jerry Cheng-Yen Lai, Fang-Ming Hwang, Li-Yin Chien

**Affiliations:** 1 School of Nursing, College of Medicine, National Taiwan University, Taipei, Taiwan. Address: No.1, Sec. 1, Ren'ai Rd., Zhongzheng Dist., Taipei City 10051, Taiwan; 2 Department of Nursing, National Taiwan University Hospital, Taipei, Taiwan. Address: No.7, Chung Shan S. Rd., Zhongzheng Dist., Taipei City 10002, Taiwan; 3 Department of Medical Research, Taitung MacKay Memorial Hospital, Taitung, Taiwan. Address: 1, Lane 303, Changsha Street, Taitung 95054, Taiwan; 4 Department of Education, National Chiayi University, Chiayi, Taiwan. Address: No.300, Syuefu Rd., Chiayi City 60004, Taiwan; 5 Institute of Community Health Care, School of Nursing, National Yang-Ming University, Taipei, Taiwan. Address: No.155, Sec. 2, Linong St., Beitou Dist., Taipei City 11221, Taiwan

**Keywords:** Acculturation, bi-dimensional acculturation, childbirth, depression, immigrant, pregnancy

## Abstract

**Background:**

Childbirth may pose many challenges to the psychological well-being of marriage-based immigrant mothers in interracial marriages, who must negotiate bi-dimensional acculturation – adaptation to the host culture and maintenance of her own heritage culture. We examined the temporal relationships between bi-dimensional acculturation and depressive symptoms from pregnancy to 1 year postpartum among marriage-based immigrant mothers in Taiwan using the cross-lagged structural equation modeling.

**Methods:**

This study recruited 310 immigrant mothers, who were examined in the second and third trimesters, and again at 1 month, 3 months, 6 months, and 1 year postpartum from March 2013 to December 2015. Depressive symptoms and bi-dimensional acculturation were measured using the Edinburgh Postnatal Depression Scale and Bidimensional Acculturation Scale for Marriage-Based Immigrant Women, respectively.

**Results:**

The study found that adaptation to the host culture followed a downward linear trajectory, while maintenance of the mother's own heritage culture followed an upward linear trajectory from pregnancy to 1 year postpartum. All but one cross-lagged path between bi-dimensional acculturation and depressive symptoms was statistically insignificant, though almost all cross-sectional associations were significant. Adaptation to host culture was negatively associated with depressive symptoms at all time points. The association between maintenance of heritage culture and depressive symptoms reversed from positive to negative after 6 months postpartum.

**Conclusions:**

Adaptation to the host culture and maintenance of the mother's heritage culture differed in their associations with maternal depressive symptoms. Health professionals should assist immigrant mothers in adapting to the host culture while supporting their heritage culture in the childbearing period.

## Introduction

Transnational marriage has been increasing around the world (Charsley, Bolognani, Spencer, Ersanilli, & Jayaweera, 2016; United Nations, 2016). During the past three decades, nearly one-tenth of all marriages in Taiwan were between marriage-based immigrant women and Taiwanese men (Department of Household Registration, 2019). Most marriage-based immigrant women in Taiwan are from Southeast Asian countries, with the majority from China (67%) and Vietnam (21%) (National Immigration Agency, 2018). Taiwan is a modern Chinese society with a patrilineal culture. In Chinese culture, children are seen as an extension of the family line and pregnancy/childbirth is the key for a wife to integrate into the husband's family (Hsia, 2000; Liu, Chung, & Hsu, 2001). For marriage-based immigrant women, the perinatal period is challenging as they become pregnant soon after their arrival in Taiwan, and the need to acquire maternal roles and adapt to new family relationships (Liu et al., 2001; Yang & Wang, 2003). Chinese culture has special perinatal customs, including specific rules, behavioral restrictions, task assistance, and care for pregnant women and new mothers. According to traditional Chinese custom, women should remain at home, follow restrictive rules and receive assistance with practical tasks for at least 1 month after birth, which is commonly referred to as ‘doing the month’ (Chen, Tai, Wu, Chiang, & Chien, 2012; Chien, Tai, Ko, Huang, & Sheu, 2006). Marriage-based immigrant women in Taiwan are usually expected to adapt the mainstream Taiwanese perinatal cultural practices of their spouses and in-laws, which may differ from their original cultural practices (Chen, Tai, et al., 2012).

Acculturation is a multidimensional process and a confluence of heritage-cultural and receiving-cultural practices, values, and identifications (Schwartz, Unger, Zamboanga, & Szapocznik, 2010). Acculturation can be broadly categorized as unidimensional or bi-dimensional. Unidimensional acculturation involves adaptation only to the host country. This focus on cultural adaptation is based on the perception that immigrants must spend more time in the host society and become more oriented toward the host culture while relinquishing their own cultural heritage (Cuellar, Harris, & Jasso, 1980; Gordon, 1964; Marin & Sabogal, 1987). Alternatively, psychologists have proposed a bi-dimensional acculturation model to emphasize the importance of both heritage and host cultures and enhance cultural integration (Berry, 1980; Bourhis, Moise, Perreault, & Senecal, 1997).

Acculturation has been considered a critical sociocultural predictor of health-related outcomes in immigrant populations (Lommel & Chen, 2016; Ro, 2014). However, few studies have explored the relationship between bi-dimensional acculturation and depression during pregnancy or the postpartum period (Abbott & Williams, 2006; Jackson et al., 2015; Walker, Ruiz, Chinn, Marti, & Ricks, 2012). Walker et al. (2012) indicated that a low level of adaptation to the host culture was a significant risk factor for prenatal depression among Hispanic immigrant women between 22 and 24 weeks' gestation in the USA; however, there was no statistically significant relationship between the level of maintenance of the heritage culture and prenatal depression. Abbott and Williams (2006) reported that a low level of maintenance of the heritage culture was significantly associated with an increased risk of depression at 6 weeks postpartum among women who immigrated to New Zealand from the Pacific Islands, but the level of adaptation to the host culture was not significantly related to postpartum depression. Jackson et al. (2015) revealed no significant correlations between Mexican/Anglo American orientation and prenatal/postpartum depression. There have been discordant results concerning the relationship between bi-dimensional acculturation and perinatal depression.

Fuligni (2001) indicated that a major limitation of the acculturation literature is cross-sectional assessment. Dynamic acculturation trends might best be assessed over a long or sensitive period of cultural transformation. However, only one study has examined the bi-dimensional acculturation trajectory in the postpartum period (Schluter, Tautolo, & Paterson, 2011). A New Zealand study of immigrant mothers revealed that adaptation to the host culture linearly increased with the number of years spent living in New Zealand. With regard to the maintenance of the mother's heritage culture, the level remained almost constant among postpartum mothers who had lived in New Zealand for under 12 years while there was a linear decline among those who had lived in New Zealand for more than 12 years (Schluter et al., 2011).

Overall, little is known about the bi-dimensional acculturation and depression trajectories from pregnancy to the postpartum period as well as the temporal relationship between acculturation and depression among immigrant women, especially in non-Western populations. To our knowledge, no existing studies have examined the effect of acculturation on marriage-based immigrant women. Since the spouse and in-laws are from a different culture, marriage-based immigrant women are highly susceptible to bi-dimensional acculturation during perinatal period. Due to the ever increasing numbers of marriage-based immigrant women worldwide, there is therefore a strong incentive to investigate the dynamic acculturation trends during a culturally sensitive period, such as the perinatal period in the Chinese culture, to provide further understanding on the effect of cultural transformation on psychological health. Therefore, the aim of this study was to explore the bi-dimensional acculturation and depressive symptom trajectories from pregnancy to the postpartum period and to examine their relationships in marriage-based immigrant women in Taiwan. The research questions were: (1) What is the trajectory of bi-dimensional acculturation and depressive symptoms from pregnancy to 1 year postpartum? (2) What is the temporal relationship between bi-dimensional acculturation and perinatal depressive symptoms?

## Methods

### Design and procedure

In this prospective longitudinal study, women were recruited to complete structured questionnaires in the second and third trimesters of their pregnancy as well as at 1 month, 3 months, 6 months, and 1 year postpartum. The data collection period was March 2013 to December 2015.

We cooperated with health centers, clinics, and hospitals to recruit pregnant immigrant women. Those who agreed to participate signed an informed consent form and provided their contact information. The study protocol was approved by the institutional review boards of Mackay Memorial Hospital, Tzu Chi General Hospital Taipei Branch, Taipei City Hospital, and Saint Mary's Hospital Luodong.

The participants completed the study questionnaires either through face-to-face interviews, telephone interviews, by post, or e-mail based on their preferred mode of data collection. If participants did not reply within 2 weeks of the indicated follow-up time, their second preference with regard to mode of communication was used for data collection. Participants received a voucher worth about 100 New Taiwan dollars (1 US$ = 29 NTD in August 2020) as a token of gratitude.

### Participants

The inclusion criteria were adult pregnant immigrants who were born outside of Taiwan and married to Taiwanese men, were more than 12 weeks pregnant, and currently lived in Taiwan. A total of 372 pregnant immigrant women were referred to the research team, of whom 62 (16.7%) refused to participate in the study. The final sample for this analysis included 310 participants recruited during pregnancy (184 women recruited in the second trimester and 126 in the third trimester); 142 (77.2%) of the 184 women recruited in the second trimester were interviewed again in the third trimester. Of the 310 participants, 191 (61.6%), 175 (56.5%), 166 (53.5%), and 209 (67.4%) completed the follow-up interviews at 1 month, 3 months, 6 months, and 1 year postpartum, respectively. Immigrant women who contributed data did not differ from those that did not contribute data at each time point in age, educational level, employment status, and family income (online Supplementary Table S1).

### Sample size considerations

To achieve a power of 0.8, effect size of 0.1, *α* of 0.05, and number of measurements as 6, at least 109 participants are needed for repeated measure design using G-Power software. Therefore, the sample size was sufficient.

### Measures

The study variables included sociodemographic, obstetric, and immigration-related factors, personal history of psychiatric disease, bi-dimensional acculturation and depressive symptoms. Bi-dimensional acculturation and depressive symptoms were time-variant variables, while the remainder were time-invariant variables. Depression was measured at all time points, while bi-dimensional acculturation was measured during pregnancy as well as at 3 months, 6 months, and 1 year postpartum.

The immigration-related factors consisted of birth country, age at immigration, duration of living in Taiwan, and Chinese language ability. Chinese language ability was measured using a four-item self-report tool for listening, speaking, reading, and writing ability scored on a five-point Likert scale from 0 (very poor) to 4 (very good). The scale has been used in previous studies on immigrant women in Taiwan (Chen et al., 2013b; Chen, Hsu, Chu, Han, & Chien, 2012; Chen, Hwang, Tai, & Chien, 2013a).

Depression was assessed using the Edinburgh Postnatal Depression Scale (EPDS), which had been used and validated in women of various cultural backgrounds (Gibson, McKenzie-McHarg, Shakespeare, Price, & Gray, 2009; Murray & Cox, 1990). The EPDS is a 10-item instrument intended to assess perceived depressive symptoms over the previous 7 days (Cox, Holden, & Sagovsky, 1987). Scored on a four-point Likert scale, it has been widely used to screen for depressive symptoms and measure their severity, with scores ranging from 0 to 30. The EPDS score can be used as a continuous variable, with higher scores indicating higher levels of depressive symptoms (Chen et al., 2013*a*, *b*; Chen et al., 2016).

As no bi-dimensional acculturation scales were developed and validated among marriage-based immigrant women, bi-dimensional acculturation was assessed using the self-developed Bidimensional Acculturation Scale for Marriage-Based Immigrant Women (BAS-MBIW), which included adaptation to the host culture and maintenance of the heritage culture. The BAS-MBIW was measured using two parallel 19-item, five-point Likert and six-domain scales (language use, media use, food preference and use, cultural heritage, social interaction, and goods preference and use) with a possible range of 0–76. High scores on specific subscales indicate a higher level of adaptation to the host culture or a higher level of maintenance of the heritage culture. Expert review and exploratory factor analysis of the BAS-MBIW indicated acceptable content validity and construct validity (online Supplementary material and online Supplementary Table S2). The internal consistency for adaptation to the host culture was 0.88, and for maintenance of the heritage culture was 0.83, as assessed by the Cronbach's *α* coefficient.

### Data analysis

The study variables were described using percentages, means, and standard deviations. Trajectories of bi-dimensional acculturation and depression were examined using the null generalized estimating equation (GEE) model with only the linear and/or quadratic term for time in the model.

Bivariate correlation between bi-dimensional acculturation and depression during pregnancy and at 3 months, 6 months, and 1 year postpartum were assessed using the Pearson's correlation. The interrelationships between bi-dimensional acculturation and depressive symptoms were explored by the cross-lagged approach using structural equation modeling (SEM; Maruyama, 1998). SEM produced parameter estimates for each path loading. A value <5 for χ^2^/df, a value >0.8 for the normed fit index (NFI) and the comparative fit index (CFI), and a value <0.1 for the root-mean-square error approximation (RMSEA) signal good model fit. Statistical significance was determined by a two-sided *p* value <0.05.

We used available case analysis because Little's Missing Completely at Random (MCAR) test yielded a *p* of >0.05, indicating that the data missing was at random (Li, 2013). Therefore, we ignored missing and the analytical sample size was 310 during pregnancy (184 for the second trimester and 268 for the third trimester), 191 at 1 month, 175 at 3 months, 166 at 6 months, and 209 at 12 months postpartum.

The data were analyzed using IBM SPSS Statistics 22.0 (IBM Corp., Armonk, NY, USA). SEM was analyzed using Amos 23.0 (IBM Corp., Chicago, IL, USA).

## Results

### Characteristics of the participants

The characteristics of the participants are summarized in Table 1. The mean age of the immigrant women was 29.94 years (s.d. = 4.41; range = 20.42–43.92). About 15% of the women reported insufficient family income, 1.6% of the women reported having been diagnosed with a psychiatric disease. Approximately two-thirds of the immigrant women came from China and the remaining one-third came from Southeast Asia and other countries. The mean age at immigration was 26.24 years (s.d. = 4.50). The mean EPDS score was 4.11 (s.d. = 5.06) in the second trimester, 4.24 (s.d. = 4.72) in the third trimester, 5.57 (s.d. = 5.78) at 1 month postpartum, 6.09 (s.d. = 6.05) at 3 months postpartum, 6.92 (s.d. = 6.54) at 6 months postpartum, and 5.33 (s.d. = 5.97) at 1 year postpartum.

**Table 1.** Characteristics of the study participants: time-invariant variables (*N* = 310)

Variables	*N*	%	Mean	s.d.
Sociodemographic variables				
Age			29.94	4.41
<25 y	39	12.6		
⩾25 y	271	87.4		
Educational level^a^				
Senior high school or lower	204	66.0		
University or higher	105	34.0		
Employment status				
Unemployed	220	71.0		
Employed	90	29.0		
Family income				
Insufficient	44	14.2		
Just making a living	141	45.5		
Sufficient	125	40.3		
Obstetric variables				
Parity				
1	169	54.5		
>1	141	45.5		
Complications during pregnancy^b^				
No	235	79.1		
Yes	62	20.9		
Delivery complications^c^				
No	234	86.0		
Yes	38	14.0		
Fetal/Infant sex^d^				
Girl	139	46.2		
Boy	162	53.8		
History of psychiatric disease (including depression)				
No	305	98.4		
Yes	5	1.6		
Immigration-related characteristics				
Birth country				
China	212	68.4		
Southeast Asian and other countries^#^	98	31.6		
Age at immigration^e^			26.24	4.50
Duration of living in Taiwan (year)^e^			3.66	4.32
Chinese language ability^a^			13.58	3.92

*Note:* ^a^*n* = 309; ^b^*n* = 297; ^c^*n* = 272; ^d^*n* = 301; ^e^*n* = 308. ^#^Vietnam: 50 (16.1%); Myanmar: 14 (4.5%); Indonesia: 13 (4.2%); Malaysia: 7 (2.3%); Philippines: 4 (1.3%); Thailand: 2 (0.6%); Cambodia: 2 (0.6%); Others: 6 (2.0%). Complications during pregnancy included antepartum hemorrhage, gestational hypertension, and gestational diabetes mellitus. Delivery complications included laceration, postpartum hemorrhage, infection, fever, anesthesia-related complications, and amniotic fluid embolism.

### Trajectories of bi-dimensional acculturation and depressive symptoms

**Fig. 1.**
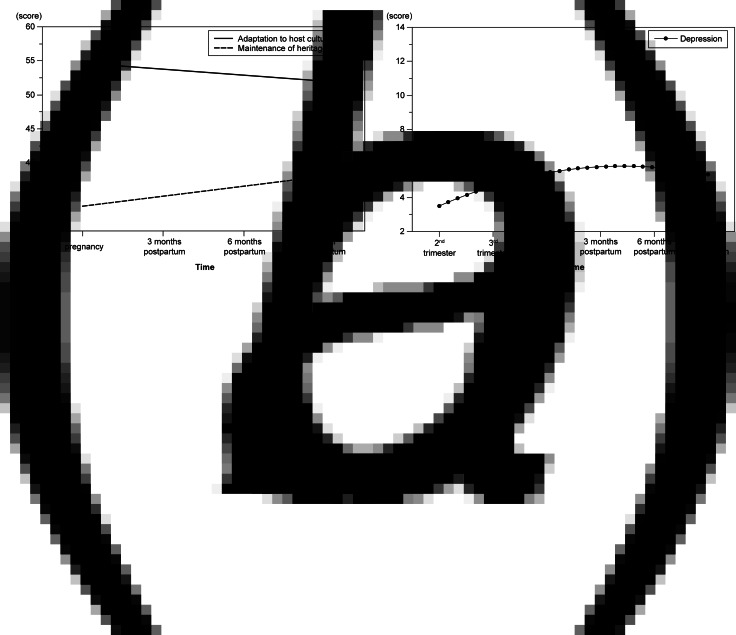
Time trends of bi-dimensional acculturation and depression from the second trimester to 1 year postpartum.

In the GEE null models, adaptation to the host culture followed a downward linear trajectory (linearly decreased with time), whereas maintenance of the heritage culture followed an upward linear trajectory (linearly increased with time). In the null models of depression, the linear and quadratic terms were both significant. Depression followed an upward curvilinear trajectory such that depression scores were lowest in the second trimester, which then increased sharply until 3 months postpartum, flattened out between 3 and 6 months postpartum, and gradually decreased between 6 months and 1 year postpartum. See Table 2 and Fig. 1.

**Table 2.** Null model of trajectories from the second trimester to 1 year postpartum

Variables	Acculturation		Depression *β* (s.e.)
	Adaptation to host culture *β* (s.e.)	Maintenance of heritage culture *β* (s.e.)	
Linear model			
Intercept	55.53 (0.77)**	32.17 (0.82)**	3.70 (0.32)**
Linear	−0.88 (0.16)**	1.47 (0.18)**	0.37 (0.08)**
Quadratic model			
Intercept			1.93 (0.67)**
Linear			1.77 (0.47)**
Quadratic			−0.20 (0.06)**

s.e., standard error.

*Note*: Working correlation matrix: AR(1). **p < 0.01.

### Bi-dimensional acculturation and depressive symptoms

Bivariate correlation among adaptation to host culture, maintenance of heritage culture, and depressive symptoms during pregnancy, at 3 months, 6 months, and 1 year postpartum is presented in Table 3. Depressive symptoms, adaptation to host culture, and maintenance of heritage culture were each correlated with the same measures at different time points.

**Table 3.** Correlation coefficients among adaptation to host culture, maintenance of heritage culture, and depressive symptoms during pregnancy and in the postpartum period

	Adaptation to host culture				Maintenance of heritage culture				Depression			
	Pregnancy	3 months postpartum	6 months postpartum	1 year postpartum	Pregnancy	3 months postpartum	6 months postpartum	1 year postpartum	Pregnancy	3 months postpartum	6 months postpartum	1 year postpartum
Adaptation to host culture												
Pregnancy	1											
3 months postpartum	0.45***	1										
6 months postpartum	0.370***	0.52***	1									
1 year postpartum	0.32***	0.44***	0.47***	1								
Maintenance of heritage culture												
Pregnancy					1							
3 months postpartum					0.38***	1						
6 months postpartum					0.36***	0.52***	1					
1 year postpartum					0.32***	0.34***	0.45***	1				
Depressive symptoms												
Pregnancy	−0.23***	−0.14	−0.20*	−0.06	0.16**	0.09	0.12	−0.05	1			
3 months postpartum	−0.14^a^	−0.27**	−0.34***	−0.13	−0.01	0.05	−0.04	−0.14	0.54***	1		
6 months postpartum	−0.05	−0.29**	−0.36***	−0.08	−0.04	−0.08	−0.13	−0.11	0.44***	0.65***	1	
1 year postpartum	−0.14^b^	−0.08	−0.18*	−0.20**	0.05	0.19*	−0.07	−0.20**	0.40***	0.52***	0.52***	1
Mean	54.55	51.26	50.95	50.01	33.76	39.62	39.93	41.06	4.18	6.09	6.92	5.33
s.d.	11.655	12.763	12.260	10.332	12.196	13.783	13.372	12.858	4.719	6.054	6.543	5.970

*p < 0.05; **p < 0.01; ***p < 0.001.

a *p* = 0.061; ^b^*p* = 0.052 (a borderline significance).

Adaptation to host culture and depressive symptoms was negatively correlated throughout perinatal period (during pregnancy: *r* = −0.23, *p* < 0.001; at 3 months: *r* = −0.27, *p* < 0.01; 6 months: *r* = −0.36, *p* < 0.001; 1 year: *r* = −0.20, *p* < 0.01). In addition, adaptation to host culture and subsequent depressive symptoms were negatively correlated (*r* = 0.14, *p* = 0.061 for adaptation to host culture during pregnancy and depressive symptoms at 3 months; *r* = −0.29, *p* < 0.01 for adaptation to host culture at 3 months and depressive symptoms at 6 months; *r* = −0.18, *p* < 0.05 for adaptation to host culture at 6 months and depressive symptoms at 1 year). Depressive symptoms at 3 months were negatively correlated with adaptation to host culture at 6 months postpartum (*r* = −0.34, *p* < 0.001).

Maintenance of heritage culture was positively correlated with depressive symptoms during pregnancy and at 3 months postpartum, but the correlation turned negative at 6 months and 1 year postpartum. The concurrent correlations between maintenance of heritage culture and depressive symptoms were only significant during pregnancy (*r* = 0.16, *p* < 0.01) and at 1 year postpartum (*r* = −0.20, *p* < 0.01). No significant correlation between maintenance of heritage culture and subsequent depressive symptoms or *vice versa* was found.

### Cross-lagged SEM model on the temporal relationship between bi-dimensional acculturation and depressive symptoms

**Fig. 2.**
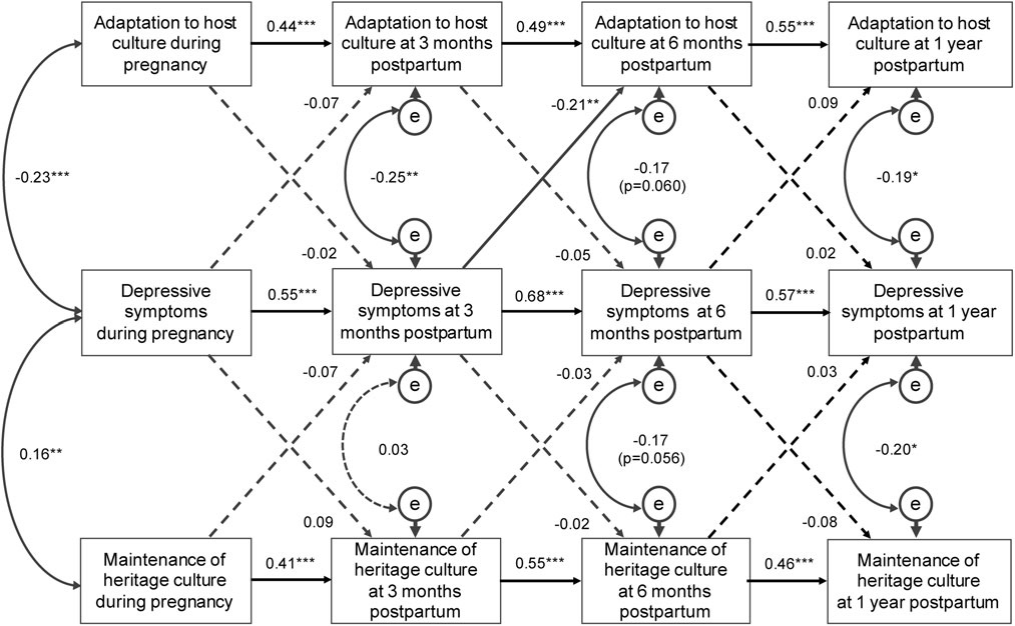
Structural equation modeling linking bi-dimensional acculturation and depression during pregnancy, at 3 months, 6 months, and 1 year postpartum. Data presented were path coefficients. A dashed line shows that the indicated path is not statistically significant. *p < 0.05; **p < 0.01; ***p < 0.001.

The interrelationship among bi-dimensional acculturation and depressive symptoms during pregnancy, at 3 months, 6 months, and 1 year postpartum was examined by SEM (Fig. 2). Only one cross-lagged path from depressive symptoms at 3 months to the adaptation to host culture at 6 months was statistically significant (coefficient = −0.21, *p* < 0.01), all other cross-lagged paths were not statistically significant. Depressive symptoms, adaptation to host culture, and maintenance of heritage culture were each directly and positively associated with those at the next time point.

Adaptation to host culture during pregnancy and depressive symptoms during pregnancy was negatively correlated (−0.23, *p* < 0.001) and the negative association was consistently present through time (coefficient = −0.25, *p* = 0.003 at 3 months, coefficient = −0.17, *p* = 0.060 at 6 months, coefficient = −0.19, *p* = 0.013 at 1 year postpartum).

Maintenance of heritage culture and depressive symptoms was negatively correlated during pregnancy (coefficient = 0.16, *p* < 0.001) and at 3 months postpartum (coefficient = 0.03, *p* = 0.728); however, they were positively correlated at 6 months (coefficient = −0.17, *p* = 0.056) and 1 year (coefficient = −0.20, *p* = 0.012) postpartum.

The fit indices suggested a good fit for both models (model fit of adaptation to host culture and depressive symptoms: χ^2^/df = 3.64; NFI = 0.89, CFI = 0.92, RMSEA = 0.092; model fit of maintenance of heritage culture and depressive symptoms: χ^2^/df = 3.66; NFI = 0.88, CFI = 0.90, RMSEA = 0.093).

## Discussion

### Bi-dimensional acculturation trajectories

The study found that adaptation to the host culture decreased, while maintenance of the heritage culture increased linearly from pregnancy to 1 year postpartum. Since the Chinese culture values pregnancy and childbirth, concurrent with the host culture, immigrant women may receive increased assistance and care from their husbands' families throughout pregnancy and early postpartum. After ‘doing the month’ (usually 1–2 months postpartum), the additional assistance and care fades, immigrant mothers need to take care of their newborns on their own, and in addition, take on an increasing number of household tasks. During this period, immigrant mothers tend to approach their original families or friends and use familiar Internet platforms or interfaces commonly used in their original countries for assistance and information. Therefore, adaptation to host culture peaked during pregnancy and ‘doing the month’ period then decreased over time; while maintenance of heritage culture was lowest in pregnancy and ‘doing the month’ period but increased over time.

Our findings differed from those in previous studies with increased adaptation to host culture and decreased maintenance of heritage culture over time for non-perinatal immigrant women (Miller, Wang, Szalacha, & Sorokin, 2009; Tseng, Wright, & Fang, 2015). In addition, the trajectory of adaptation to the host culture showed a linear decrease over time in this study, which differed from the linear growth trajectory found in a previous study of postpartum immigrant women in the Western context (Schluter et al., 2011). Schluter et al. (2011) reported a piecewise trend in the maintenance of heritage culture in New Zealand; postpartum mothers who had spent fewer than 12 years in New Zealand maintained their heritage culture, whereas those who had spent more than 12 years in New Zealand exhibited a linear decrease in the maintenance of the heritage culture. We noted that past studies did not examine marriage-based immigrant women, and the differences could be due to differences in the populations studied. Nevertheless, the observed discrepancies may have been due to different sociocultural backgrounds, measurement tools, time points of measurements, and follow-up periods among studies.

### Trajectory of depressive symptoms

Depression scores were the lowest in the second trimester, increased sharply until 3 months postpartum, flattened out between 3 and 6 months postpartum, and gradually decreased between 6 months and 1 year postpartum. Depression scores were significantly higher in the postpartum period than during pregnancy. The increase in depressive symptoms before 1 month postpartum could be due to changes in hormone levels (Bloch, Daly, & Rubinow, 2003; Yim, Tanner Stapleton, Guardino, Hahn-Holbrook, & Dunkel Schetter, 2015), and the increase in depressive symptoms between 1 and 6 months postpartum may be due to an increase in maternal tasks and parenting stress (Vismara et al., 2016). As immigrant mothers become more familiar with parenting and return to normal life (Chen et al., 2016), depressive symptoms slowly decrease from 6 months to 1 year postpartum.

### Bi-dimensional acculturation for depressive symptoms

This study is unique in exploring temporal relationship between bi-dimensional acculturation and depressive symptoms using cross-lagged SEM. However, the cross-lag temporal relationship between bi-dimensional acculturation and depressive symptoms was mostly not significant in the SEM model, suggesting a lack of direct causal relationship at the selected time points. The only significant cross-lagged path was depressive symptoms at 3 months postpartum to adaptation to host culture at 6 months postpartum. The results suggest that health professionals could develop strategies to decrease depressive symptoms at early postpartum, and which could later result in an increased level of adaptation to host culture among immigrant mothers. Given that the SEM cross-lagged path model is just one approach to explore possible causal relationship, a different time interval schedule of follow-up and experimental study may be needed to further examine the causal relationships.

Adaptation to the host culture and depressive symptoms were negatively associated throughout perinatal period. In other words, a higher level of adaptation to the host culture was associated with decreased depression scores from pregnancy to 1 year postpartum among marriage-based immigrant women in Taiwan. This negative association has previously been reported in an American study (Walker et al., 2012).

The direction of correlations between maintenance of heritage culture and depressive symptoms gradually changed from positive correlations during pregnancy and at 3 months postpartum to negative correlations at 6 months and 1 year postpartum. A higher level of maintenance of the heritage culture was associated with increased depression scores from the second trimester to 3 months postpartum; however, a higher level of maintenance of the heritage culture was associated with decreased depression scores from 6 months to 1 year postpartum. The effect of maintenance of the heritage culture on maternal depression differed by time; this result was different from a New Zealand study reporting a negative association at 6 weeks postpartum (Abbott & Williams, 2006). The discordance may be because, in this study, immigrant women who had a high level of maintenance of their heritage culture risked conflict between the perinatal cultures of the host and heritage countries and could experience difficulties because of the absence of a friendly environment to maintain their heritage culture, and this could potentially lead to feelings of depression during pregnancy and the early postpartum period. Three months postpartum, postpartum cultural ‘doing the month’ practices faded and family assistance with tasks ended. To care for themselves and their babies, immigrant women actively seek help from resources of their heritage culture. Therefore, a higher level of maintenance of the heritage culture could lead to decreased depressive symptoms in the late postpartum period.

Bivariate correlations between adaption to host culture and subsequent depressive symptoms were not shown in the SEM model, suggesting the observed correlations may not be real and could be due to the strong carryover effect of depression. Depressive symptoms and bi-dimensional acculturation were each correlated with the subsequent time point, suggesting relatively strong stability in the variables across time. Taken together, cross-sectional but non-causal associations were found between bi-dimensional acculturation and perinatal depressive symptoms among marriage-based immigrant women. The lack of causal relationship could be due to time points selected. For instance, acculturation level may be established soon after arrival at the immigrant country and only changed to a little later. Nonetheless, the cross-sectional associations suggest the importance of incorporating bi-dimensional acculturation into programs targeting perinatal depressive symptoms among immigrant mothers.

### Limitations

This is the first longitudinal study that attempts to understand the effect of bi-dimensional acculturation on depression from pregnancy to the postpartum period. However, some limitations of this study should be mentioned. First, to increase the sample size and variations in the desired cohort, we conducted convenience sampling and recruited participants from clinics, different hospital levels, and health centers. However, we were unable to access the information of those who refused to participate in the study. The comparison of age of the parturient of live births between our study participants and all immigrant mothers using the national birth reporting system showed no statistical difference between the two groups (Health Promotion Administration, 2014). To increase the response rate in the follow-up period, all possible modes of data collection were applied. However, attrition rates were still relatively high, potentially leading to selection bias. Depressive symptoms were measured using a self-administered questionnaire. The possibility of endogeneity and reverse causality cannot be excluded despite the study's longitudinal design. Lastly, the study participants were marriage-based immigrant mothers, whether the results could be generalized to different types of immigrant mothers merit further studies.

## Conclusions

For marriage-based immigrant mothers, adaption to the host culture and maintenance of the mother's heritage culture are dynamic and time-variant during pregnancy and the postpartum period. Higher levels of adaptation to host culture were associated with lower levels of perinatal depressive symptoms. Higher maintenance of the heritage culture was associated with higher levels of perinatal depressive symptoms from pregnancy to 3 months postpartum, and higher maintenance of the heritage culture was associated with lower levels of postpartum depressive symptoms from 6 months to 1 year postpartum. Adaptation to the host culture and maintenance of the mother's heritage culture differed in their impact on maternal depressive symptoms. To decrease perinatal depressive symptoms and benefit bi-dimensional acculturation, health professionals should assist immigrant mothers in adapting to the host culture while supporting their own culture, and resolving potential cultural conflicts in the childbearing period.

## Supplementary material

For supplementary material accompanying this paper visit https://doi.org/10.1017/S0033291720004195. click here to view supplementary material
