# Supplementary material for: Bi-dimensional acculturation and depressive symptom trajectories from pregnancy to 1 year postpartum in marriage-based immigrant women in Taiwan
Source: Psychol Med. 2020 Dec 2;52(12):2290–8. doi: 10.1017/S0033291720004195 (PMC9527671; doi:10.1017/S0033291720004195)
Supplement: Supplementary file 1 [file S0033291720004195sup001.docx]

**Supplementary material**

**Methods**

Validity of the BAS-MBIW

The content validity was examined by five experts who specialised in the fields of social science, nursing, public health, and medical professionals. The experts reviewed completeness, clarity, and consistency of each item. All experts rated the relevance of each item using the content validity index (CVI) scoring sheet with a 4-point scale (1: not relevant; 2: somewhat relevant; 3: quite relevant; 4: highly relevant). Suggestions for revisions were provided when items had a score of less than 3.

Item-level CVI (I-CVI) was computed as the number of experts rating a score of 3 or 4 then divided by the total number of experts. Scale-level CVI (S-CVI) was the content validity of the overall scale, defined as proportion of items on a scale that achieves a relevance rating of 3 or 4 by all the experts (Polit & Beck 2006). An I-CVI and S-CVI level of 80% was deemed acceptable (Polit & Beck 2006). The I-CVIs ranged from 0.6 to 1.0. The S-CVI/UAs of two scales were both 0.83. Four items with scores less than 3 were discussed with the experts and deleted. After deletion of the 4 items, the I-CVI was all 1.0 and the S-CVI was both 1.0.

Construct validity was supported by the exploratory factor analysis. The factor analysis results are shown in the Table S2.

**Reference**

Polit, D. F., & Beck, C. T. (2006). The content validity index: are you sure you know what's being reported? Critique and recommendations. *Research in Nursing & Health, 29*, 489-497.

Table S1. The comparison between the participants with complete data and those that did not complete data during the third trimester, at 1 month, 3 months, 6 months, and 1 year postpartum (N=310)

| Sociodemographic variables | 3^rd^ trimester | | | 1 month postpartum | | | 3 months postpartum | | | 6 months postpartum | | | 12 months postpartum | | |
| --- | --- | --- | --- | --- | --- | --- | --- | --- | --- | --- | --- | --- | --- | --- | --- |
|  | Complete  (n=268) | Not complete  (n=42) |  | Complete  (n=191) | Not complete  (n=119) |  | Complete (n=175) | Not complete  (n=135) |  | Complete  (n=166) | Not complete  (n=144) |  | Complete  (n=209) | Not complete  (n=101) |  |
|  | n (%) | n (%) | *p* | n (%) | n (%) | *p* | n (%) | n (%) | *p* | n (%) | n (%) | *p* | n (%) | n (%) | *p* |
| Age |  |  | 0.23 |  |  | 0.95 |  |  | 0.67 |  |  | 0.14 |  |  | 0.87 |
| <25 y | 37 (13.8%) | 2 (4.8%) |  | 25 (13.1%) | 14 (11.8%) |  | 24 (13.7%) | 15 (11.1%) |  | 27 (16.3%) | 12 (8.3%) |  | 28 (13.4%) | 11 (10.9%) |  |
| ≥25 y, <30y | 113 (42.2%) | 16 (38.1%) |  | 79 (41.4%) | 50 (42.0%) |  | 71 (40.6%) | 58 (43.0%) |  | 62 (37.3%) | 67 (46.5%) |  | 84 (40.2%) | 45 (44.5%) |  |
| ≥30 y, <35y | 81 (30.2%) | 18 (42.8%) |  | 62 (32.4%) | 37 (31.1%) |  | 53 (30.3%) | 46 (34.1%) |  | 54 (32.5%) | 45 (31.3%) |  | 68 (32.5%) | 31 (30.7%) |  |
| ≥35 y | 37 (13.8%) | 6 (14.3%) |  | 25 (13.1%) | 18 (15.1%) |  | 27 (15.4%) | 16 (11.8%) |  | 23 (13.9%) | 20 (13.9%) |  | 29 (13.9%) | 14 (13.9%) |  |
| Educational level^a^ |  |  | 0.25 |  |  | 0.47 |  |  | 0.83 |  |  | 0.22 |  |  | 0.49 |
| Senior high school or lower | 173 (64.8%) | 31 (73.8%) |  | 129 (67.5%) | 75 (63.6%) |  | 114 (65.5%) | 90 (66.7%) |  | 114 (69.1%) | 90 (62.5%) |  | 140 (67.3%) | 64 (63.4%) |  |
| University or higher | 94 (35.2%) | 11 (26.2%) |  | 62 (32.5%) | 43 (36.4%) |  | 60 (34.5%) | 45 (33.3%) |  | 51 (30.9%) | 54 (37.5%) |  | 68 (32.7%) | 37 (36.6%) |  |
| Employment status |  |  | 0.05 |  |  | 0.64 |  |  | 0.51 |  |  | 0.64 |  |  | 0.33 |
| Unemployed | 196 (73.1%) | 24 (57.1%) |  | 132 (69.1%) | 88 (74.0%) |  | 122 (69.7%) | 98 (72.6%) |  | 121 (72.9%) | 99 (68.8%) |  | 143 (68.4%) | 77 (76.2%) |  |
| Part-time employment | 16 (6.0%) | 6 (14.3%) |  | 14 (7.3%) | 8 (6.7%) |  | 15 (8.6%) | 7 (5.2%) |  | 12 (7.2%) | 10 (6.9%) |  | 17 (8.2%) | 5 (5.0%) |  |
| Full-time employment | 56 (20.9%) | 12 (28.6%) |  | 45 (23.6%) | 23 (19.3%) |  | 38 (21.7%) | 30 (22.2%) |  | 33 (19.9%) | 35 (24.3%) |  | 49 (23.4%) | 19 (18.8%) |  |
| Family income |  |  | 0.95 |  |  | 0.48 |  |  | 0.56 |  |  | 0.63 |  |  | 0.88 |
| Insufficient | 38 (14.2%) | 6 (14.3%) |  | 24 (12.6%) | 20 (16.8%) |  | 28 (16.0%) | 16 (11.9%) |  | 22 (13.2%) | 22 (15.3%) |  | 30 (14.4%) | 14 (13.9%) |  |
| Just making a living | 121 (45.1%) | 20 (47.6%) |  | 91 (47.6%) | 50 (42.0%) |  | 79 (45.1%) | 62 (45.9%) |  | 73 (44.0%) | 68 (47.2%) |  | 93 (44.5%) | 48 (47.5%) |  |
| Sufficient | 109 (40.7%) | 16 (38.1%) |  | 76 (39.8%) | 49 (41.2%) |  | 68 (38.9%) | 57 (42.2%) |  | 71 (42.8%) | 54 (37.5%) |  | 86 (41.1%) | 39 (38.6%) |  |

*Note:* ^a^n = 309

*p* values were based on *X*^2^ tests.

Table S2. Exploratory factor analysis of the adaptation to the host culture and maintenance of the heritage culture subscales in the Bi-dimensional Acculturation Scale for Marriage-Based Immigrant Women (BAS-MBIW)

|  | Item | Adaptation to the host culture subscale | | | Maintenance of the heritage culture subscale | | |
| --- | --- | --- | --- | --- | --- | --- | --- |
|  |  | Factor loading | Eigen  value | % of variance | Factor loading | Eigen  value | % of variance |
| Language use | |  | 2.5 | 13.13 |  | 4.9 | 25.95 |
|  | How often do you speak Mandarin or Taiwanese/your native language with adult family members? | 0.95 |  |  | 0.96 |  |  |
|  | How often do you speak Mandarin or Taiwanese/your native language with young family members? | 0.93 |  |  | 0.96 |  |  |
|  | How often do you speak Mandarin or Taiwanese/your native language with neighbors, friends, or co-workers? | 0.94 |  |  | 0.95 |  |  |
|  | How often do you use Mandarin or Taiwanese/your native language to think/memorize? | 0.84 |  |  | 0.93 |  |  |
| Media use | |  | 1.5 | 8.14 |  | 1.8 | 9.36 |
|  | How often do you watch local/your mother country’s TV channels or listen to local/your mother country’s radio stations? | 0.67 |  |  | 0.85 |  |  |
|  | How often do you read local/your mother country’s newspapers, magazines or books? | 0.89 |  |  | 0.90 |  |  |
|  | How often do you sing in local/your mother country’s dialects? | 0.88 |  |  | 0.84 |  |  |
| Food preference and use | |  | 1.9 | 10.09 |  | 1.4 | 7.51 |
|  | How often do you enjoy local/your mother country’s cuisine? | 0.67 |  |  | 0.72 |  |  |
|  | How often do you choose local/your mother country’s cuisine when you dine out? | 0.89 |  |  | 0.87 |  |  |
|  | How often do you eat local/your mother country’s food at home? | 0.88 |  |  | 0.86 |  |  |
| Cultural heritage | |  | 1.8 | 9.32 |  | 2.3 | 12.16 |
|  | How often do you follow Taiwanese/your mother country’s traditional rituals? | 0.80 |  |  | 0.92 |  |  |
|  | How often do you enjoy Taiwanese/your mother country’s festivals? | 0.88 |  |  | 0.94 |  |  |
|  | How often do you accept Taiwanese/your mother country’s folk beliefs? | 0.89 |  |  | 0.92 |  |  |
| Social interaction | |  | 1.2 | 6.37 |  | 1.3 | 6.64 |
|  | How often do you interact with friends who are native Taiwanese/from your mother country? | 0.97 |  |  | 0.97 |  |  |
|  | How often do you ask friends who are native Taiwanese/from your mother country for opinions or assistance? | 0.97 |  |  | 0.97 |  |  |
| Goods preference and use | |  | 6.6 | 34.59 |  | 4.0 | 21.14 |
|  | Do you prefer shopping in a store owned by a person who is Taiwanese/from your mother country? | 0.95 |  |  | 0.89 |  |  |
|  | How often do you shop in a store owned by a person who is Taiwanese/from your mother country? | 0.97 |  |  | 0.89 |  |  |
|  | Do you prefer using manufactured goods that are Taiwanese/from your mother country? | 0.95 |  |  | 0.90 |  |  |
|  | How often do you choose to purchase manufactured goods that are Taiwanese/from your mother country? | 0.90 |  |  | 0.90 |  |  |
| Cumulative % of variance | |  |  | 81.64 |  |  | 82.77 |

*Note.* The underlined section refers to the part that differs between the adaptation to the host culture and maintenance of the heritage culture subscales. For the adaptation to the host culture subscale, the word or phrase before the **/** is the item. For the maintenance of the heritage culture subscale, the word or phrase that follows the / is the item.

Each item was rated on a 5-point Likert scale from 0-4 for rarely, seldom, sometimes, often, and usually, respectively.
